# Correction: 3D modeling of vector/edge finite element method for multi-ablation technique for large tumor-computational approach

**DOI:** 10.1371/journal.pone.0316568

**Published:** 2024-12-26

**Authors:** Gangadhara Boregowda, Panchatcharam Mariappan

There are errors in the captions of Figs 3, 6, 7, 8, 9 and 10. Please see the complete, correct Figs 3, 6, 7, 8, 9 and 10 captions here.

**Fig 3:**
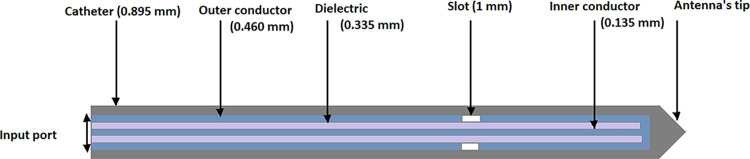
Antenna Geometry.

**Fig 6:**
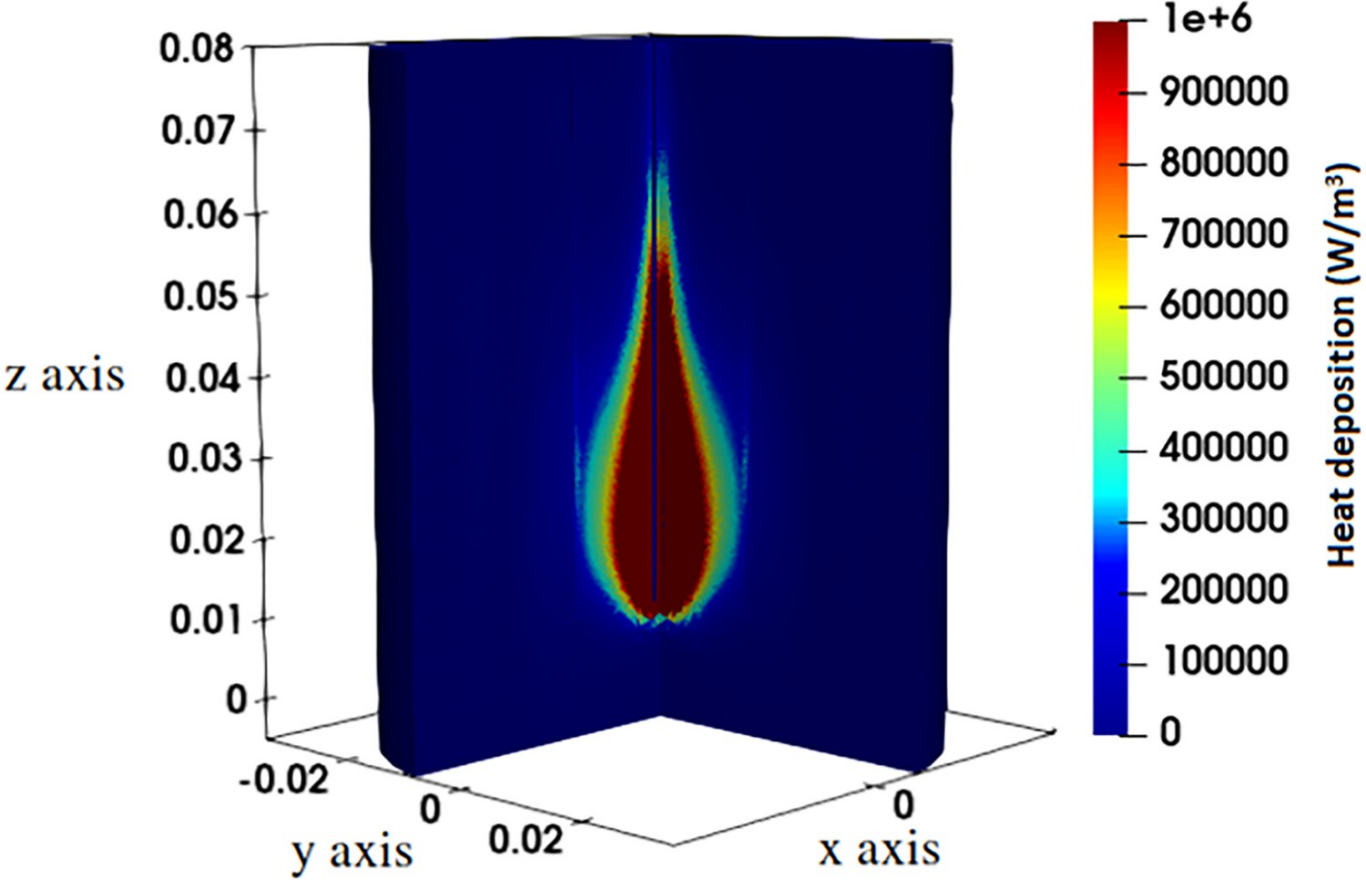
The microwave heat deposition (W/m^3^) within the liver tissue at position *P*_*1*_ using input power 50 W and frequency 2.45 GHz.

**Fig 7:**
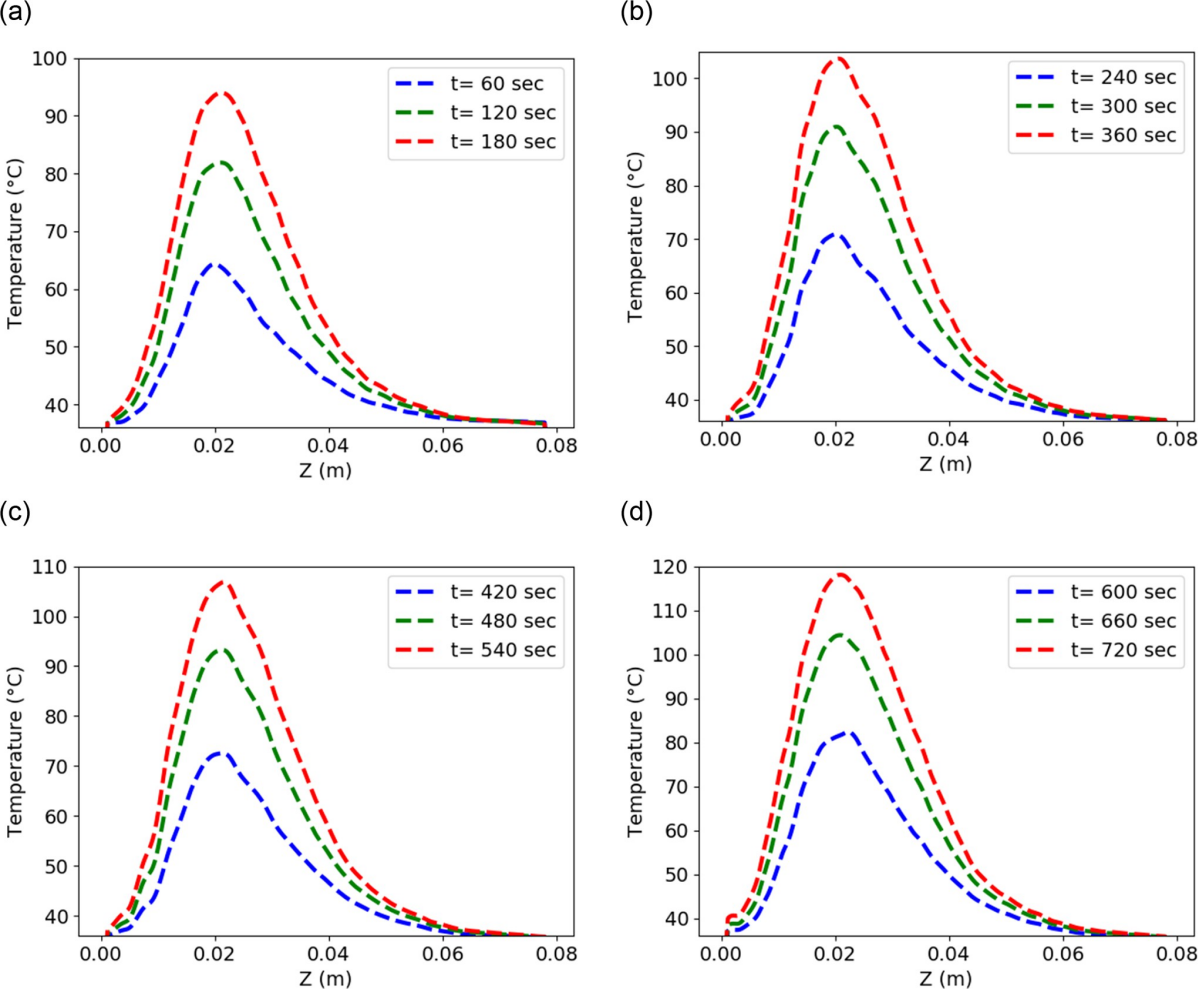
Temperature distribution using input power 50 W and frequency of 2.45 GHz in the liver along the line parallel to the antenna at (a) position *P*_*1*_ (b) position *P*_*2*_ (c) position *P*_*3*_ (d) position *P*_*4*_.

**Fig 8:**
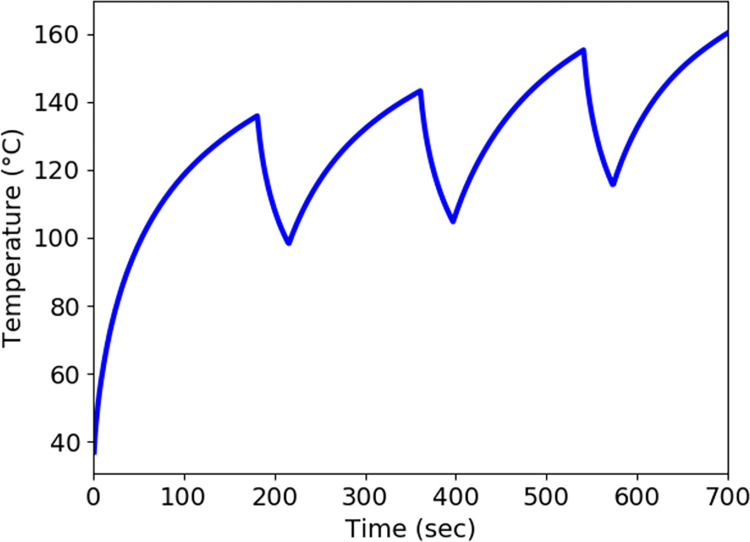
Temperature profile near the slot in the liver at input power 50 W and frequency of 2.45 GHz during the treatment.

**Fig 9:**
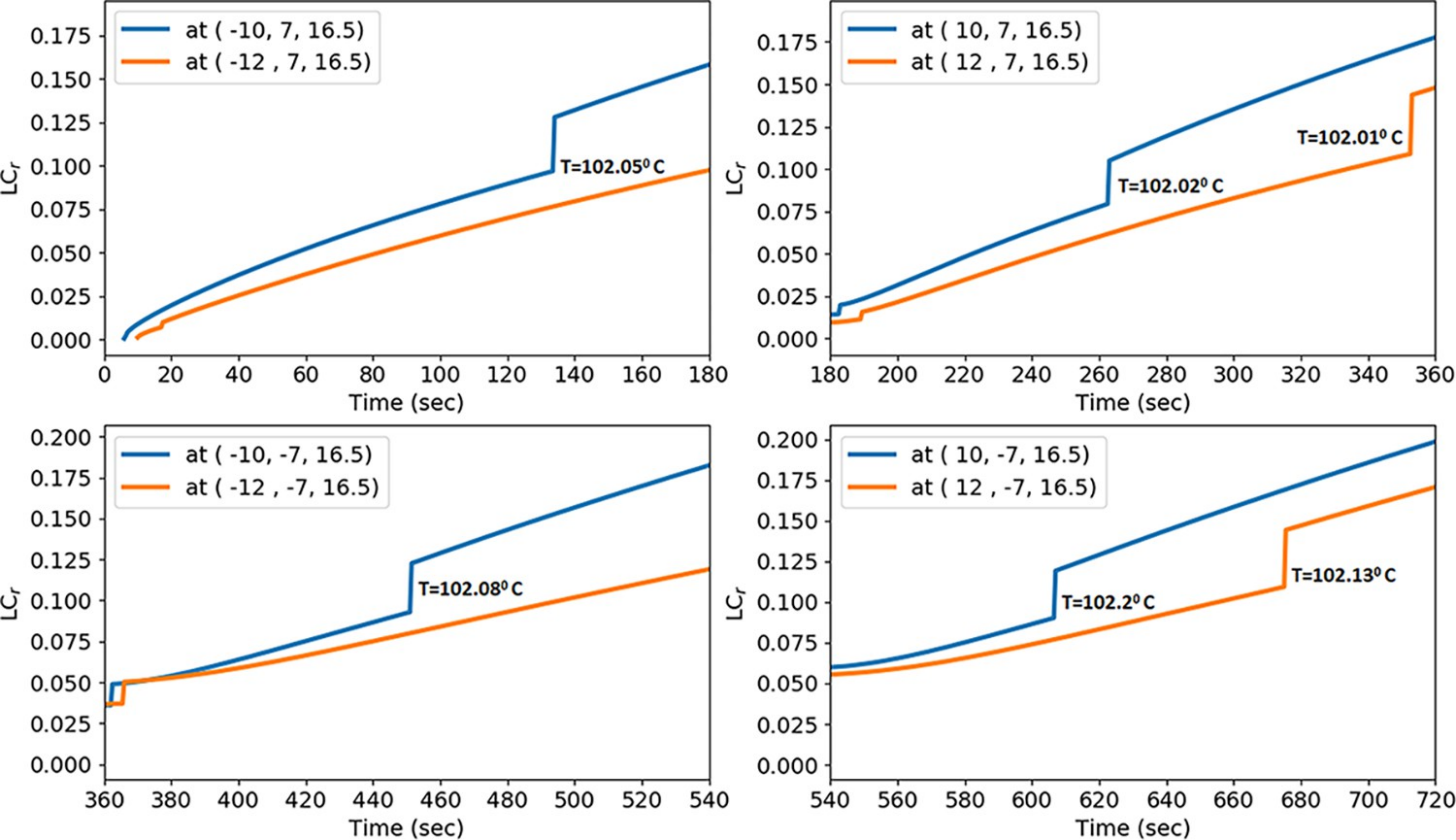
Localized contraction at microwave power 50 W at 3 mm and 5 mm away from the position *P*_*1*_,*P*_*2*_, *P*_*3*_, and *P*_*4*_ for time intervals [0, 180], [180, 360], [360, 540], and [540, 720], respectively.

**Fig 10:**
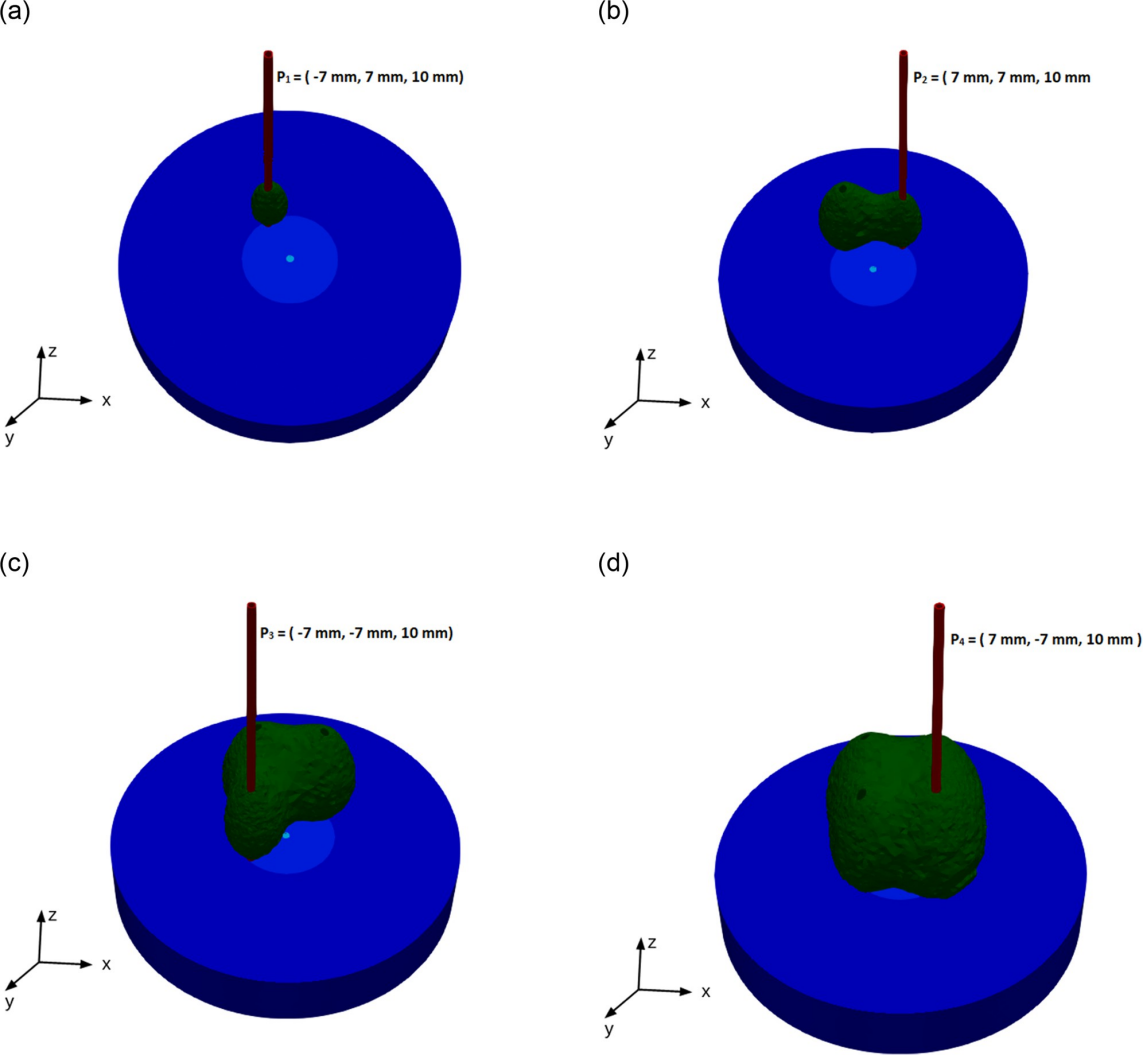
Ablation zone at (a) t = 180 sec (b) t = 360 sec (c) t = 540 sec (d) 720 sec.
