# Supplementary material for: 3D modeling of vector/edge finite element method for multi-ablation technique for large tumor-computational approach
Source: PLoS One. 2023 Jul 28;18(7):e0289262. doi: 10.1371/journal.pone.0289262 (PMC10381062; doi:10.1371/journal.pone.0289262)
Supplement: S1 File — (PDF) [file pone.0289262.s002.pdf]

Supporting information

S1 Appendix. Implementation of FEM for unsteady bioheat equation. By multiplying the bioheat equation by a scalar test function and integrating over the domain Ω, the equation is converted to a variational form.

$$\rho C \int_{\Omega} \frac{\partial T}{\partial t} V d\Omega = -k \int_{\Omega} \nabla T \cdot \nabla V d\Omega + \int_{\Omega} Q_e V d\Omega + (\rho_b C_b \omega_b) \int_{\Omega} (T_b - T) V d\Omega + \int_{\Omega} Q_m V d\Omega$$

where V is a scalar test function.

Let $\mathbb{V}_h \subset H_0^1(\Omega)$ be a finite element space with basis functions $\{\phi_1, \phi_2, ..., \phi_N\}$. Semi discretized finite element method is to find $T_h(t) \in \mathbb{V}_h$ for any $t > 0$ such that

$$\rho C \int_{\Omega} \frac{\partial T_h}{\partial t} V_h d\Omega = -k \int_{\Omega} \nabla T_h \cdot \nabla V_h d\Omega + \int_{\Omega} Q_e V_h d\Omega + (\rho_b C_b \omega_b) \int_{\Omega} (T_b - T_h) V_h d\Omega + \int_{\Omega} Q_m V_h d\Omega \tag{25}$$

Since $\mathbb{V}_h$ is spanned by basis functions $\{\phi_1, \phi_2, ..., \phi_N\}$. One can express $T_h = \sum_{i=1}^{N} T_i(t)\phi_i(x)$, where N is the number of nodes and coefficient $T_i(t)$ is functional value of $T(x,t)$ at node i. By using the Galerkin approach, Eq (25) is transformed into a system of equations.

$$\rho C B \dot{T}(t) + kAT(t) + \rho_b C_b \omega_b BT(t) = F(t)$$

where $\dot{T}(t) = \frac{\partial T(t)}{\partial t}$

where $B = (b_{ij}),\ A = (a_{ij}),\ F = (F_i),\ T = (T_i)$

$$\begin{aligned} b_{ij} &= \int_{\Omega} \phi_i \phi_j d\Omega \\ a_{ij} &= \int_{\Omega} \nabla \phi_i \cdot \nabla \phi_j d\Omega \\ F_i &= \int_{\Omega} (Q_e + \rho_b C_b \omega_b T_b + Q_m) \phi_i d\Omega \end{aligned}$$

Discretize the time interval $(0, T)$ into a uniform grid with step size Δt. Let $t^n = n\Delta t$ for $n = 0, 1, 2, ..M$.

$$[\rho C B + kA\Delta t + \rho_b C_b \omega_b B \Delta t] T^n = [\rho C B] T^{n-1} + \Delta t F^n$$

After obtaining the stiffness matrices for each element, the stiffness matrices were assembled using FEniCS[1], and the system of equations was then solved using the Multifrontal Massively Parallel Sparse Direct Solver (MUMPS).

S2 Appendix. Implementation of FDM for cell-death model The first-order difference formula yields the following result as an approximation of the time derivative of the fractions of alive and dead cells.

$$\frac{dA}{dt} = \frac{A^{n+1} - A^n}{\Delta t} \tag{26}$$

$$\frac{dD}{dt} = \frac{D^{n+1} - D^n}{\Delta t} \tag{27}$$

where $A^n = A(x, t_0 + n\Delta t)$ and $D^n = D(x, t_0 + n\Delta t)$ are fraction of alive and dead cells at time $t = t_n$

By substituting Eqs (26) and (27) in the cell death model, one can calculate fractions of alive and dead cells at $(n+1)^{\text{th}}$ time step with initial value $A^0 = 0.99$ and $D^0 = 0$, as follows.

$$\begin{pmatrix} 1 + \Delta t(k_f)_{n+1} + k_b\Delta t & \Delta t k_b \\ \Delta t(k_f)_{n+1} & 1 + \Delta t(k_f)_{n+1} \end{pmatrix} \begin{pmatrix} A^{n+1} \\ D^{n+1} \end{pmatrix} = \begin{pmatrix} \Delta t k_b + A^n \\ \Delta t(k_f)_{n+1} + D^n \end{pmatrix}$$

Reference

1. Alnæs MS, Logg A, Mardal KA, Skavhaug O, Langtangen HP. Unified framework for finite element assembly. International Journal of Computational Science and Engineering. 2009;4(4):231–244.
