# Supplementary material for: 3D modeling of vector/edge finite element method for multi-ablation technique for large tumor-computational approach
Source: PLoS One. 2023 Jul 28;18(7):e0289262. doi: 10.1371/journal.pone.0289262 (PMC10381062; doi:10.1371/journal.pone.0289262)
Supplement: Attachment — Submitted filename: Response to Reviewers.odt [file pone.0289262.s003.odt]

Response to the Reviewer #1

1)The novelty of the paper should be clarified in the abstract and conclusion.
ANS: Thank you for the suggestion, and modified it according to that.

2)Authors should include some other qualitative and quantitative results in the abstract.
ANS: Thank you for the suggestion, and modified it according to that.

3)The overview in the introduction is not complete. It is necessary to review the literature and supplement some relevant literature appropriately. An updated literature review should be conducted based on recent novel studies. It can be helpful to present the state-of-the-art and knowledge gaps in the research.
ANS: Thank you for the suggestion, and we tried our best to improve the literature review.

4)Some of your equations don't have a prestigious reference.
ANS: We verified the references and gave proper references from prestigious articles

5)Validation should be performed in a specific new section.
ANS:  Thank you for the suggestion, and modified it according to that.

6)Conclusions should be stronger and arranged into a few substantive and interconnected paragraphs instead of a short lump of text. The language is deficient in places.
ANS: Thank you for the suggestion, and modified according to that.

7)There are some grammatical errors in the manuscript. Be sure to address them.
ANS:  Thank you for the suggestion, and we tried our best to reduce the manuscript's grammatical errors.


Response to the Reviewer #2


1) Within the abstract, it is suggested to include more outcomes about results here achieved in terms of ablated zones, as done by the authors within the conclusions
ANS: Thank you for the suggestion, and modified it according to that.

2) Please define the aspect ratio when introducing Table 1.
ANS:  We included the aspect ratio as the ratio of the Diameter to the Length in Table 1.

3) Please report references for the antenna geometry when introducing governing equations. Many geometries have been proposed through the years, so why did the authors decided to use exactly this antenna? Please note that ablation zones might depend on the antenna employed.
ANS:
The single-slot coaxial antenna is one of the commercial antennae which is widely used in clinical surgery. And single slot antennas can produce optimum SAR and temperature distribution in the liver. Also, this was used in one of the Author's previous projects for treating live patients, so the technical specifications and limitations of this antenna are well known, which is also the reason for using this antenna.

[1)Gas P. Study on interstitial microwave hyperthermia with multi-slot coaxial antenna. arXiv preprint arXiv:2008.02032. 2020 Aug 5.
2)Rattanadecho, Phadungsak, and Pornthip Keangin. "Numerical study of heat transfer and blood flow in two-layered porous liver tissue during microwave ablation process using single and double slot antenna." International Journal of heat and mass Transfer 58.1-2 (2013): 457-470.]

We are aware that the ablation zone depends on the antenna type, and we studied the literature on antenna type and ablation zone and presented it in the manuscript. In this research, we focused on single-slot coaxial antennas for the reasons mentioned above.


4) When describing heat transfer, why did the authors decided to use Pennes bio-heat model among the ones proposed in literature through the years [5, 6]? Please report some discussion about this point here
ANS: A well-known model for examining the heat distribution in living organisms is Pennes' bio-heat equation. It is assumed that the blood perfusion effect is homogeneous and isotropic. And aslo it will not describe the effect of directional blood flow on heat distribution[1].
 We used Pennes's bio-heat model because the liver and parameters are homogeneous and isotropic, and Blood vessels and blood flow were not considered in the computational domain[1-2] . 
 One has to introduce the convection term due to blood flow to the bio-heat equation to analyze blood flow. Klinger et al.[2] developed the model to study directional blood flow by introducing convection terms in the bio-heat equation.

[[1]Keangin P, Rattanadecho P. Analysis of heat transport on local thermal non-equilibrium in the porous liver during microwave ablation. International Journal of Heat and Mass Transfer. 2013 Dec 1;67:46-60.
[2]Tehrani MH, Soltani M, Kashkooli FM, Raahemifar K. Use of microwave ablation for thermal treatment of solid tumors with different shapes and sizes—A computational approach. PLoS One. 2020 Jun 15;15(6):e0233219.
[3]Peng T, O'Neill DP, Payne SJ. A two-equation coupled system for determination of liver tissue temperature during thermal ablation. International Journal of Heat and Mass Transfer. 2011 Apr 1;54(9-10):2100-9.
]
5) Did the authors here neglect perfusion dependence from the temperature [7]? If so, please report this as an assumption for the present computations.
ANS: Since we did not consider the blood vessels in the computation domain, a simple  On/OFF blood perfusion model was used in this work[1]. Since microvasculature collapses when the temperature exceeds 60 degrees, we assumed the perfusion rate for each element was constant until the temperature crossed the threshold of 60 degrees, after which it was set to 0 for the remainder of the simulation (representing complete vascular coagulation).  

[[1]Schutt DJ, Haemmerich D. Effects of variation in perfusion rates and of perfusion models in computational models of radio frequency tumor ablation. Medical physics. 2008 Aug;35(8):3462-70.
[2]Trujillo M, Berjano E. Review of the mathematical functions used to model the temperature dependence of electrical and thermal conductivities of biological tissue in radiofrequency ablation. International Journal of Hyperthermia. 2013 Sep 1;29(6):590-7.]

6) In the reviewer's opinion, novelty from the present paper could be better described by improving the state of art in the introduction. Indeed, there are many contributions from the literature referred to the last years that haven't been considered in the literature review, with references to both modeling approach and novel solutions for MW ablation. Since in this contribution, the authors focused on both aspects, some improvements in the manuscript are suggested. Microwave ablation have been extensively analyzed by including different aspects within the predictive models such as variable local properties, tissue dilation, as well as tissue shinkrage ; on the other hand, with references to the ablation procedures, pulsating heat sources, or number of slots employed, have been proposed through the years. Therefore, in order to underline what has been done up to now in the open literature, what should be done, and what is done here, the authors should improve their state of art based on these observations.
ANS: Thank you for the suggestion, and we tried our best to improve the literature review.

7) A cell death model is presented here in a separate subsection. Why did the authors decide to use this approach instead of other ones, say CEM43, Arrenhius damage model, or temperature threshold criterion? Please report here the pro and cons of the present approach selected for the cell death model
ANS: The most simple models use a single temperature threshold above which cells are instantaneously declared dead and below which cells remain fully functional. These isotherm models take no account of the duration for which cells are at elevated temperature despite it being known that this factor influences the cellular response. Since these models do not consider the transition state between the alive and dead states, it is difficult to analyze the thermal damage accurately.
 The two main limitations of  Arrehnius-based models are[1]: 1) The Arrehnius parameters (frequency parameter (1/s) and activation energy (J/mol))  are highly sensitive to experimental values. 2) The cell survival rates for different temperatures show a marked discontinuity at temperatures around 43 °C, the exact temperature break depending upon species of origin and cell line. Although limitations exist with Arrhenius-based models, it has become the yardstick to which other models are compared and contrasted.
 The CEM43 °C is also an Arrhenius-based model; the parameter RCEM the CEM43 °C model depends much more strongly on activation energy than temperature[1-2]. After observing the breakpoint in the Arrhenius plot at 43 °C, by default, this model assumes 43 °C as the reference point for thermal damage.
 To overcome the above-mentioned limitation of the cell response model, O'Neill et al. (2011) suggested a cell death model by introducing a vulnerable state between the alive and dead states[2]. The parameter in the model is constructed from an exponential curve fit. Moreover, this model exhibits the "shoulder region"  found in experimental data. The model can predict fast cell death as a function of temperature during thermal treatments and can account for the further slow death of cells in the days following treatments. Since the model consists of a system of ODEs, one has to choose the correct numerical scheme with the proper time step to get an accurate solution. An implementation may be computationally high compared to other cell response models.

The following literature explains more clearly the above-mentioned points.
[[1]Pearce JA. Comparative analysis of mathematical models of cell death and thermal damage processes. International Journal of Hyperthermia. 2013 Jun 1;29(4):262-80
[2]Van Rhoon GC, Samaras T, Yarmolenko PS, Dewhirst MW, Neufeld E, Kuster N. CEM43° C thermal dose thresholds: a potential guide for magnetic resonance radiofrequency exposure levels?. European radiology. 2013 Aug;23:2215-27.
[3]O'Neill DP, Peng T, Stiegler P, Mayrhauser U, Koestenbauer S, Tscheliessnigg K, Payne SJ. A three-state mathematical model of hyperthermic cell death. Annals of biomedical engineering. 2011 Jan;39:570-9.
]


8)It is not really clear how did the authors decided to locate the antenna within the liver. Which criterion did they use? As future plans, they could also report that some optimization of this procedure might be used by employing different location, timing, and positions, for the antenna. Optimizing ablation shape is still a challenging task to obtain the best ablated zone [13], and using this procedure might be really helpful.
ANS: The spherical shape is a common shape covering most of the tumor shapes [1]. Therefore, in this work, the tumor is modeled as a sphere with a diameter of 4 cm. One of the aims of this work is to create a rounded ablation zone using multi-ablative techniques. Hence, we chose the location of the antennas in a square pattern so that heat is distributed uniformly in the spherical tumor. The separation distance between the antennas was selected based on the literature[2]. The Author suggested that if the separation distance exceeds 17 mm, overall lession may form less rounded, and there may be a chance to survive tumor cells between the two antennas. Therefore, for the safer side, we choose a separation distance of 14 mm between the two antennas to avoid the above-mentioned problems. We did not observe clefts in the ablation zone when we used 14 mm as the separation distance. One has to take care while choosing the separation distance and heating time based on the tumor size. The relationship between the separation distance, heating time, and tumor size for multi-ablative techniques is left for future investigation.

[[1]Tehrani MH, Soltani M, Kashkooli FM, Raahemifar K. Use of microwave ablation for thermal treatment of solid tumors with different shapes and sizes—A computational approach. PLoS One. 2020 Jun 15;15(6):e0233219.
[2]Wright AS, Lee FT, Mahvi DM. Hepatic microwave ablation with multiple antennae results in synergistically larger zones of coagulation necrosis. Annals of Surgical Oncology. 2003 Apr;10:275-83.]


9) Model validation is here shown with two different values of the r-coordinate. Are the experimental results [14] from ex-vivo or in-vivo experiments? Furthermore, even if the agreement looks fairly well, could the authors report some discussion about why for r = 4.5 mm, the model underpredicts temperatures, while for r = 7 mm there is some overprediction?
ANS: Yes, the numerical solution from the present study was measured at two positions (4.5 mm and 7 mm) and compared with the experimental data at the same positions, which was obtained by Yang et al.  At present, we are not able to answer why models underpredict and overpredict experimental data at 4.5 mm and 7 mm, respectively. A certain amount
of mismatch between experiment and simulation is expected as
the blood vessels and blood flow are not considered in the computation. 

[Yang D, Converse MC, Mahvi DM, Webster JG. Expanding the bioheat equation to include tissue internal water evaporation during heating. IEEE Transactions on Biomedical Engineering. 2007 Jul 16;54(8):1382-8.]

10) Within the paper, it is suggested to present some discussion about pro/cons between the present solution and a conventionally-used single-antenna solution
ANS: Thank you for your suggestion and we discussed in the discussion in the manuscripts.
"To treat the majority of regular tumors with a diameter less than 3 cm, a single-ablation technique was usually sufficient, but for those highly irregular and large tumors, a single-ablation technique was not a good choice. One can use this technique for large tumors by increasing input power and heating time. As we know from the literature, increasing heating time and input power can create an excess of heat in the liver, which can damage healthy cells. The spherical shape covers most of the tumor shapes; therefore, creating an ablation zone in a spherical shape is a necessary step in the ablation technique. For most of the antenna geometry, the single-ablation technique creates an oval-shaped ablation zone. Handling single-ablative technique is easy compared to multi-ablative technique clinically. Since finding locations for antennas is not difficult, patients can recover quickly with a single insertion in the body.

The multi-ablation technique is useful for large  regular/irregular tumor with proper antenna locations and heating time. It helps us create a rounded ablation zone and control healthy cell damage. Finding the location of the antenna, the heating time at each location, and the antenna separation distance is a difficult challenge in the multi-ablation technique. One has to take care while choosing the location and antenna separation distance for a given tumor size. The relationship between the separation distance, heating time, and tumor size for the multi-ablation technique is left for future investigation.
Handling the multi-ablation technique is difficult compared to the single-ablation technique since, after every ablation new location has to find."

Supporting literature
[1) Wang T, Lu XJ, Chi JC, Ding M, Zhang Y, Tang XY, Li P, Zhang L, Zhang XY, Zhai B. Microwave ablation of hepatocellular carcinoma as first-line treatment: long term outcomes and prognostic factors in 221 patients. Scientific reports. 2016 Sep 13;6(1):1-9.
2)Tehrani MH, Soltani M, Kashkooli FM, Raahemifar K. Use of microwave ablation for thermal treatment of solid tumors with different shapes and sizes—A computational approach. PLoS One. 2020 Jun 15;15(6):e0233219.
3)Gangadhara B, Mariappan P. A vector finite element approach to temperature dependent parameters of microwave ablation for liver cancer. International Journal for Numerical Methods in Biomedical Engineering. 2023 Jan;39(1):e3661.]

11) Please improve figures. For instance, in Fig. 4, there are no axis and colorbar names here. Is this the heat generation in W/m3?
ANS:  Thank you for the suggestion, and modified it according to that.

12)In Fig. 8 the authors report temperature evolution through time. First of all please report the spatial location of the point investigated. Secondly, if the authors consider water phase change within their model, why the temperature after 100 °C still continues its growth? Some phase change might arise
ANS: Figure 8 represents the maximum temperature in the liver over the time. The maximum temperature usually occurs near the slot or tip of the antenna. Temperature easily exceeds 100 °C  and continues its growth  for 50 W input power. A phase change will not arise very near the antenna tip and slot, it can be seen in the literature[1].
[1]Yang D, Converse MC, Mahvi DM, Webster JG. Expanding the bioheat equation to include tissue internal water evaporation during heating. IEEE Transactions on Biomedical Engineering. 2007 Jul 16;54(8):1382-8.
 

13) For Fig. 10, please report units (these should be millimeters).
ANS:  Thank you for the suggestion, and modified according to that.


Response to the Reviewer #3

1) Liver tumors are highly perfused, so dynamics of blood perfusion (temperature dependent) as well as heterogeneity of blood flow within the tumor should be considered to get realistic ablation zones. This becomes more important considering the large size of the domain considered by the authors (8cm x 8cm).
ANS:  Since the first time the 3D vector finite element method was implemented in the microwave ablation technique, we considered simple geometry without blood vessels and blood flow in the geometry. For future work, we are considering the real geometry, which includes blood vessels, tumor, and the liver. The real geometry was constructed by importing the medical images to Mimics (Materialise) or a 3D slicer.

2) Currently used Eq 11 accounts for just ON/OFF of blood perfusion instead of the complete spatiotemporal perfusion dynamics and this will not provide correct ablation zones. Authors may refer to literature Soni et al 2015 Int J Hyperthermia etc.
ANS: Since we did not consider the blood vessels in the computation domain, a simple  On/OFF blood perfusion model was used in this work[1]. Since microvasculature collapses when the temperature exceeds 60 degrees, we assumed the perfusion rate for each element was constant until the temperature crossed the threshold of 60 degrees, after which it was set to 0 for the remainder of the simulation (representing complete vascular coagulation). Also, we compared the ablation zone from the current work with an ablation zone computed using the temperature-dependent blood perfusion model from the literature[2]. We observed only a slight variation in ablation volume.

[[1]Schutt DJ, Haemmerich D. Effects of variation in perfusion rates and of perfusion models in computational models of radio frequency tumor ablation. Medical physics. 2008 Aug;35(8):3462-70.
[2] Tehrani MH, Soltani M, Kashkooli FM, Raahemifar K. Use of microwave ablation for thermal treatment of solid tumors with different shapes and sizes—A computational approach. PLoS One. 2020 Jun 15;15(6):e0233219.
[3]Trujillo M, Berjano E. Review of the mathematical functions used to model the temperature dependence of electrical and thermal conductivities of biological tissue in radiofrequency ablation. International Journal of Hyperthermia. 2013 Sep 1;29(6):590-7.]

3)Numerical value of blood perfusion, considered for the simulation, could not be located in the manuscript. Kindly mention this in Table 3 along with other parameters.
ANS: The numerical value of blood perfusion and other parameter is located near the models instead of in Table 3.

4) It is not clear, whether the electro-thermal properties corresponding to the ablated region (in total computational domain) are considered for computing the next ablated region, for sequential insertion of the electrode.
ANS: Throughout the simulation, the temperature-independent parameters are considered the same in the entire domain, including the ablated region.
For temperature-dependent parameters, the magnitude of parameters considered corresponds to the threshold temperature in the ablated region. For the non-ablated area, the parameter values considered correspond to the temperature at that position.
Example: The blood perfusion rate is zero for the Ablated region.


5) Please discuss whether the perfusion is cutoff for the first ablated region for computation of the second ablation zone (overlapping of ablation zones) for the next lectrode insertion point/time step and so on.
ANS: We discussed the treatment for parameters during the simulation for ablated and non-ablated areas in above question. Blood perfusion is off if the temperature at an element  exceeds the threshold  at once during the treatment.

6) Instead of mentioning sequential insertion points/locations in the tumor, a normalized parameter (probe separation, time points) w.r.t. tumor volume may be provided to generalize the multiple elctrode insertion locations & exposure time steps based on a tumor volume.
ANS: One of the difficulties in the multi-ablation technique is choosing the antenna separation distance and heating time, which depend on tumor size. The relationship between the separation distance, heating time, and tumor size for the multi-ablation technique is left for future investigation. As a primary investigation, we considered the tumor with a regular shape and tried to show the impact of inserting the antenna sequentially in the liver during the treatment on the ablation zone.

7) Page 10, line 242 - Please justify the chosen locations for temperature measurements.
ANS: Yang et al. experimentally measured temperature at 2.5 mm, 4.5 mm, and 7 mm radially away from the antenna [1]. And it is difficult to measure accurate temperature experimentally near the antenna, as seen in the literature. Therefore, we used the same settings and 4.5 mm and 7 mm, which are sufficiently away from the antenna to validate numerical methods, parameters, and mathematical models. 

[[1]Yang D, Converse MC, Mahvi DM, Webster JG. Expanding the bioheat equation to include tissue internal water evaporation during heating. IEEE Transactions on Biomedical Engineering. 2007 Jul 16;54(8):1382-8.]

8)Page 10, line 253 - Authors mention good agreement with the published literature. But earlier it is mentioned that this literature reports for spherical shaped ablation zones. So, how the claim of spherical zones is arrived. Fig. 6b also supports oval shaped ablation zone.
ANS: Figure 6b compares the short and long axis of the ablation zone from the present study and previously published literature for the single-ablation technique. This work used the cell death model to calculate the ablation zone. The ablation zone is compared with previously published results to validate the model. Since results are available only for single-ablation techniques in the literature, we simulate the ablation zone using the single-ablation technique only for comparison. Figure 6a shows oval shape ablation zone due to the single-ablation technique. When the multi-ablation technique is used, we achieve a spherical-shaped ablation zone at the end of the treatment, as shown in Figure 10(d).

9) Table 4 - There are still remanant tumor cells for 4 minutes exposure as mentioned. Please discuss the strategy to avoid these.
ANS: The ablation zone volume in the multi-ablation technique depends on input power, heating time, and separation distance between antennas. Increasing heating time and input power can destroy complete tumor cells. Implementing pulsating power in the treatment instead of constant input optimizes healthy cell damage.

10) Please label the axes in figures 4 and 6 as well as other such figures.
AND: Thank you for the suggestion, and modified it according to that.
