# Supplementary material for: 3D modeling of vector/edge finite element method for multi-ablation technique for large tumor-computational approach
Source: PLoS One. 2023 Jul 28;18(7):e0289262. doi: 10.1371/journal.pone.0289262 (PMC10381062; doi:10.1371/journal.pone.0289262)
Supplement: Attachment — Submitted filename: Answer to the Reviewers question.docx [file pone.0289262.s004.docx]

Responses to the Reviewers’ Questions

**We would like to thank all the reviewers for their valuable suggestions. We answered all the questions and modified the manuscript according to them. As one of the reviewers suggested, we simulated and presented the results for the following models: 1) Inserting antennas at three locations sequentially in the triangle pattern 2) producing heat at one location (single-ablation technique).**

**Antenna separation distance plays an important role in creating an appropriate ablation zone. The relation between the antenna separation distance, heating time, and tumor volume for the multi-ablation technique is left for future investigation**

**Reviewer 2**

**1] The authors report that they use a single-slot commercial coaxial antenna; in the rebuttal, the authors make references to studies from (**[**doi.org/10.48550/arXiv.2008.02032**](http://doi.org/10.48550/arXiv.2008.02032)**,** [**doi.org/10.1016/j.ijheatmasstransfer.2012.10.043**](http://doi.org/10.1016/j.ijheatmasstransfer.2012.10.043)**) about this point. What did they mean when they wrote "this was used in one of the Author's previous projects for treating live patients "? It is possible for the authors to report more details about it? For instance, are they talking about some clinical practice done via single-slot antenna? Furthermore - and far more important - please be sure that in Fig. 3 all the quotations have been included. For instance, which is the axial distance between the slot and the antenna's tip? Giving appropriate quotations for the geometry is fundamental for whom wants to reproduce the same model in the future**

**ANS:** One of the authors of this manuscript used a single-slot coaxial antenna in his previous projects, which accelerated treatment in real time using a GPU. The author has used the same antenna for experiments in the lab for that project. **(**[**https://www.gosmart-project.eu/index.html**](https://www.gosmart-project.eu/index.html)**).** Author has used same antenna in their previous work (axisymmetry 2d model) to analyse the impact of parametrs and pulstaing power on ablation zone( 2023 Jan;39(1):e3661.

doi: 10.1002/cnm.3661. )

We included all the specifications and dimensions of the antenna in Figure 3, which will help to reproduce the results.

**2] -In (**[**doi.org/10.1016/j.ijheatmasstransfer.2012.10.043**](http://doi.org/10.1016/j.ijheatmasstransfer.2012.10.043)**), it is true that the authors concluded that single-slot antennas might improve maximum SAR; on the other hand, it is also reported that the volumetric SAR is higher for the double slot MCA. Therefore, the authors should include all this when describing the geometrical model here, in order to underline that, even if some aspects might be better for double-slot antennas, the authors decided to use a single-slot antenna here**

**ANS:** Thank you for your suggestions and included your points in the introduction.

**3] The authors report in their rebuttal that they decide to use Pennes' bioheat model because liver parameters are homogeneous and isotropic, while blood vessels and flow might not be considered within the computational domain. That seems unaccurate since, if one considers tissue plus blood as a heat exchanger, their heat capacities are not so different so one would expect an increase of arterial temperature within the perfusion term. The real reason of why Pennes bioheat equation might be still used nowadays, instead of** **other model like Klinger (**[**doi.org/10.1007/BF02464617**](http://doi.org/10.1007/BF02464617)**) or Chen and Holmes (**[**doi.org/10.1111/j.1749-6632.1980.tb50742.x**](http://doi.org/10.1111/j.1749-6632.1980.tb50742.x)**), is about its simplicity (**[**doi.org/10.1016/bs.aiht.2020.07.003**](http://doi.org/10.1016/bs.aiht.2020.07.003)**,** [**doi.org/10.1016/j.ijheatmasstransfer.2007.05.030**](http://doi.org/10.1016/j.ijheatmasstransfer.2007.05.030)**) - and this can be applied to the present study, but the author should report this as a limitation of the present model here.**

**ANS:** We agree with your points, and mentioned in the manuscript. In our future work, we will use the topological geometry of the liver, which includes blood vessels, updated bio-heat equations, and fluid equations to analyse the velocity of the blood.

**4] The authors done some improvements in the introduction to describe what has been done up to now, especially with references to cells death during thermal ablation (**[**doi.org/10.1007/s10439-010-0177-1**](http://doi.org/10.1007/s10439-010-0177-1)**). However, it is still not clear for readers why researchers might be still interested in improving tumor thermal ablation modeling and standards; there is no mention in the introduction about modeling for local properties to be variable (**[**doi.org/10.1016/j.cmpb.2021.106569**](http://doi.org/10.1016/j.cmpb.2021.106569)**), tissue dilation (**[**doi.org/10.1016/j.applthermaleng.2011.06.005**](http://doi.org/10.1016/j.applthermaleng.2011.06.005)**) or shinkrage (**[**doi.org/10.3109/02656736.2014.957250**](http://doi.org/10.3109/02656736.2014.957250)**), as well as standards improvements by using time-variable sources (**[**doi.org/10.1016/j.cmpb.2020.105887**](http://doi.org/10.1016/j.cmpb.2020.105887)**) or different slots (**[**doi.org/10.1016/j.ijheatmasstransfer.2012.10.043**](http://doi.org/10.1016/j.ijheatmasstransfer.2012.10.043)**). Therefore, the paper introduction might be improved to underline that it is still important to investigate tumor thermal ablation from a research point of view**

**ANS:** The literature review in the introduction was improved as much as possible by the authors. The authors include details on the mathematical model, numerical approaches, significance of the vector finite element method, frequency range, constant and time-dependent input power, antenna design, tissue deformation, and a literature review on ablation zones using single ablation. And the author also explained the difficulties in treating large tumors using single ablation or multiple antennas. Then, finally, explain the novelty and multi-ablation technique and its importance.

**5]** **The authors report that they neglect perfusion dependence vs temperature up to 60 °C. However, if one considers the perfusion rate vs temperature linear function reported in (**[**doi.org/10.1016/j.applthermaleng.2008.08.014**](http://doi.org/10.1016/j.applthermaleng.2008.08.014)**,** [**doi.org/10.1016/j.applthermaleng.2011.06.005**](http://doi.org/10.1016/j.applthermaleng.2011.06.005)**), by considering as an example 40 °C and 60 °C, perfusion rate might vary of about 25-30%. Therefore, the authors should include neglecting temperature dependence of perfusion as an assumption of their model here**

**ANS:** Yes, the cut-off blood perfusion model is an assumption of this study, which we mentioned in the manuscript as well.

**Reviewer 4**

**1].** **In the Introduction, the frequency of 2.45 GHz is a common frequency in MWA but it is not the only one. A range of frequencies is addressed in previous studies.**

**ANS:** Yes, This study is not focused on the effect of frequency on the treatment since it has been well-established in the literature. And, also we mentioned the frequency range and optimum frequency used in microwave ablation in the introduction part. We used a fixed frequency of 2.45 GHz in our work since many researchers have been used and experimental results are available in the literature for this frequency [1].

[1] Yang, D., Converse, M.C., Mahvi, D.M. and Webster, J.G., 2007. Expanding the bioheat equation to include tissue internal water evaporation during heating. *IEEE Transactions on Biomedical Engineering*, *54*(8), pp.1382-1388.

**2]**. **Please describe the novelties and specific contribution of this work in the field in more detail in the last paragraph of the Introduction.**

**ANS:** Thank you for your suggestion. We explained this in a detailed manner in the introduction.

**3]. Thermal properties of ablated zone change after MWA. Moreover, there is a time interval between changing the location of the antenna in which the temperature of the previously ablated reduces. Do the authors consider these two points? If not, how you can inform that the effect of these two parameters is negligible?**

**ANS**: The temperature in the ablated zone starts decreasing during the clinical time (the time interval required to change the antenna locations). Since clinical time is very short in reality, as a primary investigation, we assumed clinical time was zero in simulation. Moreover, the variation of temperature away from the antenna is very low; therefore, clinical time will not affect the boundary of the ablation zone.

We simulated the ablation zone for a model for 3 minutes at each position and 1 minute as clinical time, and there is not much difference in the ablation zone (volume of the ablation zone with clinical time is 21.1 cm^3^, without clinical time is 20.9 cm^3^).

**4]**. **It suggested that the authors try the other treatment durations. For example, 5 min in two sections or 4 min in 3 parts. And find the optimum value for the relation between the MWA duration and the number of positions.**

**ANS:** One of the difficulties in the multi-ablation technique is choosing the optimum value of antenna separation distance, number of antenna locations, and heating time, which depend on tumor size. One can optimize the efficiency of the mult-ablation technique by optimizing the above factors. The relationship between the separation distance, heating time, antenna locations, and tumor size for the multi-ablation technique is left for future investigation and we will communicate separately. As a primary investigation, we considered the tumor with a regular shape and tried to show the impact of inserting the antenna sequentially in the liver during the treatment on the ablation zone.

As your suggestion, we simulated and presented the result for following settings.

1) Antennas are inserted at four locations sequentially, with 3 and 4 minutes of heating time at each location.

2) Antennas are inserted at three locations sequentially in a triangle pattern, with 5 minutes of heating time at each location.

3) Antenna inserted at a single location, with heating time 20 min.

When the antenna was placed for 4 min at each position, 95.5% of the tumor cells and 1.8 cm^3^ of healthy cells were killed. 70% of cancer cells were killed when antennas were inserted at three locations sequentially with a heating time of 5 minutes at each location. The single ablation technique took 20 minutes to kill 95.97% of the tumor, and 7.76 cm^3^ of healthy cells were killed.

**5]. It suggested that the authors compare the treatment efficacy of their presented treatment strategy with the conventional MWA treatment which is applied one time**

**ANS:** We simulated results by an antenna at one position. The single-ablation technique took 20 minutes to kill 95.97% of tumors and it kills 7.76 cm^3^ healthy cells. Whereas the multi-ablation technique destroys 95.5% of tumors and kills 1.8 cm^3^ of healthy cells.

6**]. Can you find a more efficient treatment by changing and optimizing the positions of the antenna at each step?**

**ANS:** The efficiency of the ablative treatment depends on the ablation zone created at the end of the treatment with minimum thermal damage to healthy cells. The shape and volume of the ablation zone of the multi-ablation technique depend on the antenna separation distance between the antennas and the heating time at each location.

One can create a large ablation zone during the treatment by choosing the appropriate antenna separation distance and number of locations. Thermal damage can be optimised by optimising the heating time and locations of the antenna. And also, one can use the pulsating power and directed antennas to optimize the thermal damage.

As a primary investigation, we considered a regular-shaped tumor, and antennas were inserted in a square and equitriangle pattern. Optimising the number of locations, antenna separation distance, heating time, input power, and frequency is important in multi-ablation treatment to increase the efficiency of the treatment, which is kept for future investigation.

**7]**. .**It is suggested that the authors discuss tumor contraction in more detail, i.e., the effect, its importance, its impact on treatment outcomes, and its contribution to previous studies.**

**ANS:** Thank you for your suggestion. We explained this point in the section’s result and tissue localized contraction model.

**Reviewer 5.**

**1] The following references should be cited**

**─Effects of target temperature on thermal damage during temperature-controlled MWA of liver tumor, 2022
─Modeling of heat transfer distribution in tumor breast cancer during microwave ablation therapy, 2022
─Numerical analysis of human cancer therapy using microwave ablation, 2021**

**ANS:** Above mentioned papers have been cited in the proper places.

**2] How does the water contained in the body behave when the temperature exceeds 100°C?**

**ANS:** Many researchers [1, 2, 3] have studied the remaining water content in the tissue during treatment; therefore, the author has not included it in the manuscript. We studied the water content in the tissue during the treatment and presented here. We measured the water content in the tissue at four positions:

(-10, 7, 16.5), (10, 7, 16.5), (-10, -7, 16.5), and (10, -7, 16.5) which are 5 mm away from the antenna and parallel to the slot. Water vaporization in the tissue remained constant at temperatures below 80 °C. At temperatures over 80 °C, the water content begins to drop at an increasing rate to about one-half by 104 °C and exponentially decays as the temperature gets higher.

[1] Selmi, Marwa, Abdul Aziz Bin Dukhyil, and Hafedh Belmabrouk. "Numerical analysis of human cancer therapy using microwave ablation." Applied Sciences 10, no. 1 (2019): 211.

[2] Yang, Deshan, Mark C. Converse, David M. Mahvi, and John G. Webster. "Measurement and analysis of tissue temperature during microwave liver ablation." IEEE transactions on biomedical engineering 54, no. 1 (2006): 150-155

[3] Yang, Deshan, Mark C. Converse, David M. Mahvi, and John G. Webster. "Expanding the bioheat equation to include tissue internal water evaporation during heating." IEEE Transactions on Biomedical Engineering 54, no. 8 (2007): 1382-1388.

**3] Figure 8: Is the model still valid for all the temperatures?**

**ANS**: Figure 8 represents the maximum temperature in the domains, which usually occurs near the slot or tip of the antenna. The variation in temperature near the antenna is greater, as can be seen from the experimental results [1]. As we move away from the antenna, the variation in temperature is less, and it may attain equilibrium. If we measure the temperature away from the antenna, it may not follow the Sudeen variation, rapid growth, and rapid decay in temperature.
